# Underground mine rescue robotic systems: insights into human-robot information exchange

**DOI:** 10.3389/frobt.2026.1698570

**Published:** 2026-02-11

**Authors:** Roya Bakzadeh, Rana Alhaj-Bedar, Sarah Wilson, Vasileios Androulakis, Hassan Khaniani, Sihua Shao, Mostafa Hassanalian, Pedram Roghanchi

**Affiliations:** 1 Department of Mining Engineering, University of Kentucky, Lexington, KY, United States; 2 Department of STEM Education, University of Kentucky, Lexington, KY, United States; 3 Department of Experiential Engineering Education, Rowan University, Glassboro, NJ, United States; 4 Department of Mineral Engineering, New Mexico Tech, Socorro, NM, United States; 5 Petroleum Recovery Research Center, New Mexico Tech, Socorro, NM, United States; 6 Department of Electrical Engineering, Colorado School of Mines, Golden, CO, United States; 7 Department of Mechanical Engineering, New Mexico Tech, Socorro, NM, United States

**Keywords:** human factors, mine search and rescue robots, semi-structured interviews, thematic analysis, underground mine emergencies, user-centered design

## Abstract

Mine emergencies demand rapid and informed decision-making under extreme conditions, often placing personnel in life-threatening situations. Robotic assistance offers the potential to reduce unnecessary human exposure during such operations. This study examines the specific informational needs and communication preferences of mine rescue personnel for designing robotic systems for underground emergency response. A semi-structured interview was developed and conducted with ten mine rescue personnel and subject matter experts (SMEs). Responses were analyzed using thematic analysis and compared with established cognitive models to derive key design recommendations. Drawing on both field experience and hypothetical rescue scenarios, participants provided insights into key functional aspects of robotic systems, including mapping and navigation, gas detection and environmental monitoring, communication capabilities, system reliability, control, and the robot’s specific roles during operations. The qualitative data was transcribed and analyzed to identify recurring themes and critical user guidelines. The findings revealed insights into the informational and interface recommendations of rescue teams, particularly the need for real-time situational data and customizable human–robot interfaces tailored to emergency scenarios. These results expose key deficiencies in the current human–robot interaction systems and offer actionable guidance for designing robotic technologies that better align with the operational needs of experienced responders. The outcomes of this study can serve as practical guidelines for developing effective interfaces to support underground mine rescue missions.

## 1 Introduction

Underground mine emergencies, although rare, are high-consequence events in which mine rescue personnel risk their lives by entering dangerous conditions. One of the most critical applications of robotics is in mine search and rescue operations,where it may be too dangerous, or entirely impossible, for humans to intervene (Reddy et al., 2015). However, environmental factors in underground mines (e.g., dust, gas, smoke, and darkness) create a range of challenges for the design and deployment of search and rescue robots. Such challenges include navigation, localization, communication in low-light conditions, power and energy efficiency, maintenance, human-robot interaction (HRI), and strict adherence to safety constraints (Chang et al., 2022).

To motivate this work, it is important to briefly outline how underground mine search and rescue (S&R) operations are currently conducted and where information gaps arise with and without robotic support. Following an explosion, fire, roof fall, or inundation, standard protocols require establishing a fresh-air base and deploying trained mine rescue teams equipped with breathing apparatus, gas detection instruments, and lifelines into areas where conditions are uncertain and potentially lethal (U. S.Department of Labor et al., 2013; DeJonckheere and Vaughn, 2019; Kilinc et al., 2014). In the absence of robotic assistance, any attempt to verify roof stability, gas conditions, fire spread, water accumulation, or survivable routes demands that personnel physically enter contaminated, low-visibility environments with limited time windows imposed by breathing apparatus duration, heat stress, and communication constraints. As a result, command centers must make critical decisions based on intermittent verbal reports, partial maps, and limited environmental data, which makes it difficult to maintain situational awareness beyond the team’s immediate surroundings and to project how conditions may evolve over time. Robotic systems could collect more data by extending sensing and communication deeper into the mine, scouting inaccessible or high-risk areas prior to human entry, and providing safer channels for video, mapping, and gas data transmission (Reddy et al., 2015; Li et al., 2022; Zhai et al., 2020; Wang et al., 2018). At the same time, integrating robots into mine rescue introduces its own challenges, including maintaining reliable communication through rock, maneuvering within constrained geometries, ensuring intrinsic safety, and designing control and visualization interfaces that fit established mine rescue procedures and cognitive demands under stress. These complementary benefits and constraints define the current state of the art and motivate a focused examination of what information mine rescuers need from robots and how they prefer that information to be delivered.

To address these challenges, various approaches have been pursued. Competitions such as DARPA’s Subterranean Challenge (Rouček et al., 2020) and RoboCup Rescue (RoboCup Federation, 2023) have fostered advancements in robotic exploration systems by promoting improvements in capability and reliability for subterranean environments. Additionally, numerous search and rescue robots have been designed and developed specifically for underground mining applications. Notable examples include RATLER (Krotkov et al., 1994), Numbat (Ralston et al., 2001), ANDROS Wolverine (Murphy et al., 2009), Groundhog (Li et al., 2022), Cave Crawler (Zhai et al., 2020), Souryu (Arai et al., 2008), Inuktun VGTV (Casper and Murphy, 2003), WA Water Service Company Robot (Wang et al., 2018), Subterranean Robot, Leader (Ray et al., 2010), Gemini Scout (Zhao et al., 2017), MINBOT II (Wang et al., 2014), CUMT V (Zhao et al., 2017), Mobile Inspection Platform (Novák et al., 2018), and Telerescuer (Moczulski et al., 2014). These examples, spanning development years from 1994 to 2020, demonstrate the broad effort to tackle underground mine rescue challenges.

Despite this extensive progress and the diversity of robotic systems developed, current rescue robots continue to face several significant limitations, including restricted operational range, dependence on tethered communication, maneuverability, uneven floors, loose debris, and narrow passages (Bakzadeh et al., 2023). While overcoming such limitations remains a central focus of ongoing research, these efforts alone are insufficient. The success of any robotic deployment in mine rescue operations hinges not only on functionality but also on alignment with the practical realities, workflows, and decision-making processes of rescue personnel. Thus, the human element must also be carefully considered. In particular, prior knowledge of the experiences, needs, and preferences of specialized mine rescue professionals is critical. These individuals operate under intense pressure and uncertainty, so their insights are essential to inform user-centered design and ensure safe, effective deployment in high-risk scenarios (Reddy et al., 2015). Additionally, design considerations related to data visualization and situational awareness have been underscored. Effective presentation of complex data and sustaining situational awareness for human operators are considered crucial in a user-centered design. In these high-stress, low-visibility settings, rescue teams must interpret vital information in real time. Poor data visualization can impair situational awareness and diminish the overall effectiveness of rescue operations.

This research aims to ensure that robot development aligns with real-world needs by directly involving end-users in the design process, thereby enhancing communication, usability, and the overall effectiveness of robotic systems in disaster response. This research aims to ensure that robot development aligns with real-world needs by directly involving end-users in the design process, thereby enhancing communication, usability, and the overall effectiveness of robotic systems in disaster response. In this paper, the term “human–robot information exchange” refers to both information transmitted from the robot to mine rescue teams (e.g., maps, gas and environmental data, system status, alerts) and information transmitted from humans to the robot (e.g., navigation commands, autonomy boundaries, overrides, and map edits). Accordingly, the study addresses the following research question:

“How do mine rescue personnel want to exchange information and control with a search-and-rescue robot—specifically, what data do they need to receive, what inputs or commands do they wish to provide, and how should these interactions be supported through the human–machine interface?”

To answer this question, we conducted comprehensive, semi-structured interviews with experienced mine rescue personnel to gain a deeper understanding of their expectations, needs, and preferences regarding preferred robotic systems used in underground rescue missions.

Throughout this paper, we assume an operational concept in which trained mine rescue personnel and command-center staff interact directly with the robot’s interface and retain command-and-control authority over the robot’s actions, whereas other underground miners are not expected to operate or configure the system.

## 2 Background and research gap

Prior research on search-and-rescue (S&R) robots has made great strides in core capabilities (mobility, sensing, autonomy), but often with comparatively limited emphasis on human factors and user-centered interface design. From early systems like Gemini Scout and MSRBOTS (Reddy et al., 2015), which introduced tethered communication and multi-sensor payloads, to the robust autonomous multi-robot teams in the DARPA Subterranean Challenge (Rouček et al., 2020; Kulkarni et al., 2022), researchers have increasingly tackled the mechanical and algorithmic challenges of subterranean robotics. Notable contributions include CUMT-V’s successful field trials in Chinese coal mines (Brown et al., 2025), and data-efficient coordination strategies such as topological mapping under extreme bandwidth constraints (Bayer and Faigl, 2021). Ten-year longitudinal studies (Tardioli et al., 2019) and survey reviews (Bakzadeh et al., 2023) confirm steady progress in hardware and autonomy, yet also reveal that many systems still fall short in addressing human–robot interaction (HRI) and interface usability in real rescue conditions.

This oversight is echoed in broader S&R literature. Shah and Choset (Shah and Choset, 2004) emphasized that rescue robots often become impractical in real-world scenarios unless their control systems reduce operator workload under stress. Liu et al. (Liu et al., 2007) similarly argued that “operability”—ease of use—is just as important as technical performance for rescue robots to be effective. More recently, Balta et al. (Balta et al., 2017) warned that robotic tools deployed without intuitive data management systems can overwhelm human responders, and they stressed the need for presenting mission-critical information in formats that are immediately understandable. These human-factor challenges are even more acute in mining contexts, where environmental stressors like toxic gases, collapse risks, and darkness amplify the importance of effective, trustworthy information exchange. Yet few systems have been designed with miners’ workflows or decision-making needs in mind. Previous researchers have effectively used interviews with end-users to guide the design and functionality of robotic systems across various domains. Previous research has used interviews and thematic analysis to guide the development of user-centered robotic systems across diverse domains. Beer et al. interviewed older adults to inform assistive robot design for aging in place, emphasizing independence and safety (Beer et al., 2012). In industrial settings, interviews with factory workers highlighted the role of collaborative robots in improving workplace safety and job satisfaction (Baumgartner et al., 2022). Studies have also emphasized user involvement in design processes. Interviews with robot designers showed that feedback from target users shaped emotionally effective social robots (Alves-Oliveira et al., 2022), while field interviews in pharmacies led to a prototype tailored for older adults (Chiu et al., 2025). Qualitative and empirical analyses have been widely applied to deepen insights into HRI research. Seyitoğlu and Ivanov (2024) analyzed 252 studies to develop a framework for emotionally intelligent robots. Sørensen et al. (2025) identified user acceptance themes such as utility and autonomy. Interviews with Kinova® Jaco® arm users informed design improvements through themes like training and independence (Styler et al., 2025). Other studies have examined social and healthcare robotics. Søraa et al. (2021) extracted themes on desired roles and interface preferences from interviews with older adults and caregivers. Another study with acute care nursing staff identified deployment challenges and contextual constraints for hospital robots (Ohneberg et al., 2025). These studies have offered valuable insights into the functionality, utilization, and human–robot interactions of robotic systems across diverse applications.

While research and development on mine rescue robot functionality and availability have gained momentum, no studies have explored the specific needs and expectations of mine rescuers regarding these robots, despite the extreme dangers inherent in such missions. This study attempts to fill this gap by conducting qualitative research using a semi-structured interview protocol with experienced mine rescue personnel who had real mine rescue experience and represented a diverse set of companies. Interviews were chosen as the research method because they serve as valuable tools for uncovering the narrative behind a participant’s experiences, gathering detailed insights on a subject, and obtaining unforeseen types of information (DeJonckheere and Vaughn, 2019). This paper addresses that gap by explicitly integrating user feedback and cognitive models into the development of a human–machine interface for mine rescue robotics, aiming to transform the robot from a passive tool into a collaborative cognitive partner.

## 3 Methodology and methods

### 3.1 Questionnaire design and development

The development of an effective interview tool for investigating human–robot interaction in underground mine rescue operations followed a structured, three-phase process informed by established field protocols, documented disaster histories, and insights from subject matter experts. In the first phase, the field protocols from official Mine Safety and Health Administration (MSHA) and the National Institute for Occupational Safety and Health (NIOSH) rescue handbooks and training guides were analyzed to understand team roles, data needs, and decision-making processes in high-risk scenarios. Next, all the reports on historic mine disasters were reviewed to identify information gaps where robotics could enhance safety (U. S.Department of Labor et al., 2013). Following the first two steps, initial interview questions were drafted. The questionnaire went through several iterations with expert feedback to improve relevance and technical accuracy. Collaboration with SMEs ensured alignment with research goals and real-world rescue needs. The final questionnaire included both specific functionality questions and general inquiries about the role of robotics in mine rescue missions.

### 3.2 Participant recruitment

Institutional Review Board (IRB) approval was obtained prior to conducting interviews. Following IRB approval, 10 experts with 10–50 years of experience were selected. The first participant was recruited through convenience sampling, with the rest selected via snowball sampling. In snowball sampling, existing participants recommend additional individuals who meet the inclusion criteria, which is useful for reaching small, specialized populations such as experienced mine rescuers.

Participants were eligible if they met at least one of the following criteria: (i) membership in or leadership of an underground mine rescue team; (ii) responsibility for mine rescue training, emergency-response planning, or mine safety management; or (iii) specialist involvement in the selection, provision, or evaluation of mine rescue equipment and technology. Participants included superintendents, instructors, directors, safety officers, rescue trainers, and engineers from 10 companies. Notably, 7 out of 10 had direct experience with one or more mine emergencies which was a great plus. The remaining participants, while not having entered the mine during an actual disaster, had extensive involvement in mine rescue training, mock drills, and preparedness exercises, and were therefore treated as subject matter experts on how rescue operations are organized, staffed, and equipped In the United States, only a handful of underground mine disasters have occurred in the past decade, with fewer than 300 individuals participating in actual emergency responses. Given the fact that only an estimated 15% of certified mine rescuers have ever been involved in a real-world mine disaster operation, this is considered a rare qualification (Calhoun, 2022; Kilinc et al., 2014). Access to such experienced personnel has become increasingly rare due to the declining number of underground disasters, industry retirements, and stricter safety regulations that have reduced emergency incidents. Within this context, interviewing these individuals with firsthand mine disaster response experience, regardless of when that experience occurred, is highly significant. Table 1 below details about the participants.

**TABLE 1:** Interview participants’ job descriptions, years of experience, and mine rescue experience.

No.	Job description/Role	Years of experience	Real mine rescue experience
1	Training instructor	13	Yes
2	Director of mining and industrial extension	25	No
3	Rescue equipment sale manager	20	Yes
4	Company superintendent	50	Yes
5	Owner of a health and safety company	>30	Yes
6	A president at national marine corps	52	No
7	Safety officer	30	Yes
8	General engineer	13	No
9	Underground fire and rescue trainer	Not provided	Yes
10	Mining engineer	19	Yes

**FIGURE 1:**
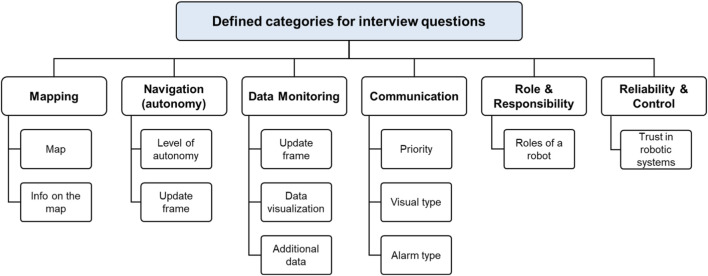
Main categories of interview questions.

Eight interviews were conducted in person and two were conducted virtually. Each interview lasted approximately 1 h and was recorded. Subsequently, the audio files were transcribed. The main emphasis was on tackling the issues concerning the accessibility and functionality of search and rescue robots, which are essential for achieving successful operations. Thus, the question “what essential capabilities do search and rescue teams expect from a robot to guarantee successful missions?” encapsulated the primary issue we were exploring. To ensure a structured and comprehensive exploration of the key considerations for the use of robots in underground mine rescue operations, the interview questions were organized around a set of predefined categories. These categories were developed based on both existing literature and preliminary consultations with SMEs, aiming to cover the full range of technical and operational concerns relevant to mine rescue scenarios. Each category reflects a core functional area in which robotic systems may support or enhance rescue efforts, including mapping, navigation, gas data monitoring, communication, robots' roles, responsibilities, and reliability and control. Within each category, specific sub-categories were identified to focus the discussion on detailed aspects such as data visualization, information update frequency, communication priorities, and interface expectations. This structured approach helped to elicit targeted feedback from participants and facilitated a consistent analysis of the interview data across different thematic areas. The categories of interview questions are shown in Figure 1 below.

### 3.3 Empirical framework for data analysis-thematic analysis

Thematic analysis has its roots in the broader field of qualitative research, with early contributions from researchers like Barney Glaser and Anselm Strauss, who developed Grounded Theory in 1967 (Glaser and Strauss, 2017). Their work on identifying recurring themes in data helped lay the foundation for thematic analysis as a distinct method. In their work, themes were systematically coded and interpreted to generate theoretical concepts, a process that later influenced the formalization of thematic analysis. Over time, this method has evolved to become a flexible and widely used technique in qualitative research, enabling researchers to identify, analyze, and interpret patterns or themes within data (Glaser and Strauss, 2017).

Thematic analysis strengthened the research approach and provided a deeper understanding by allowing a focus on identifying key patterns relevant to answering specific research questions. It goes beyond mere data summarization, as a comprehensive thematic analysis interprets the data and extracts meaningful insights (Maguire and Delahunt, 2017). By using this analytical technique, the transcripts were actively, critically, and analytically engaged with, allowing for contemplation of the significance of the data (Maguire and Delahunt, 2017). This process entailed posing questions such as: how are these experiences interpreted by the individual? What underlying beliefs are held in their understanding of their experiences? What type of reality is uncovered through their narratives?

**FIGURE 2:**
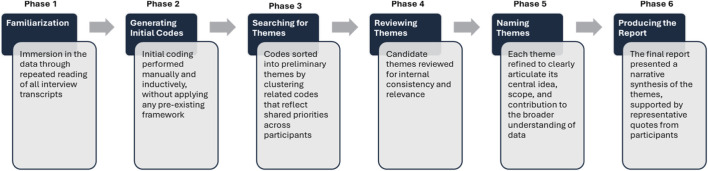
Thematic analysis phases for this study.

An inductive thematic analysis was conducted to analyze the qualitative interview data, following the six-phase guide proposed by Braun and Clarke (Braun and Clarke, 2006). This method was chosen for its flexibility and its suitability for exploring under-researched, applied contexts such as mine rescue robotics. The goal of the analysis was to identify patterns of meaning in experts’ expectations, needs, and preferences regarding the type, format, and delivery of information from underground rescue robots. This was conducted within a contextualist epistemological stance, recognizing that participants’ views are shaped both by individual experience and broader organizational or operational contexts. Figure 2 shows the detailed phases of this analysis.

### 3.4 Analytical framework for data analysis - Cognitive models

While the thematic findings offered empirical insights into user priorities, deriving evidence-based recommendations for interface design necessitated interpreting these results within the framework of established cognitive models. Cognitive models are theoretical frameworks that describe how people perceive, process, and respond to information, especially in complex, high-stress environments (Minz, 2024). In emergency response situations, mine rescuers must make rapid decisions based on partial, evolving data, often under pressure, with limited visibility, and high risk. Cognitive models allow us to predict how users will interpret information, where errors or overload may occur, and how interfaces can be designed to align with users’ mental expectations, attention limits, and decision-making processes. By applying these models, one can design robots and interfaces that are not only technically functional but also cognitively supportive, reducing confusion, building trust, and improving mission success.

For this study, four cognitive models were deliberately selected based on their relevance to the cognitive demands of rescue operations. Once the interview transcripts had been coded, themes refined, and subthemes defined, we systematically revisited each theme and its representative participant excerpts to interpret them through the lens of the four selected cognitive models. This was done using concise operational definitions for each model. Situational Awareness (SA) was included to ensure that the perception, comprehension, and projection of mine critical environmental information (Endsley, 1995). Multiple Resource Theory (MRT) was incorporated to guide the distribution of information across sensory channels, thereby reducing cognitive interference during multitasking conditions (Wickens, 2002; Wickens et al., 2021). Cognitive Load Theory (CLT) was applied to limit unnecessary mental effort and improve the clarity of information presentation, enhancing decision-making in high-pressure contexts (Sweller, 1988; Sweller et al., 2011).

Mental Models (MM) were considered to ensure that system behaviors align with user expectations, enabling more intuitive interaction and reducing the risk of error (Norman, 2002). Although additional cognitive frameworks exist, these four were selected due to their established applicability in high-risk, time-sensitive, and interface-driven environments. Collectively, they provide a theoretical foundation for developing a cognitively compatible interface for mine rescue scenarios.

## 4 Results

### 4.1 Thematic analysis results

The transcripts were reviewed alongside notes taken during data collection to ensure a deep understanding of the content and context. Initial coding was performed manually and inductively, without applying any pre-existing framework. Initially, the data was independently coded by two researchers, with interpretations drawn based on individual perspectives. When discrepancies arose, discussions were held, and a consensus was ultimately reached on the final set of codes. Seventy-five codes were generated directly from the data, focusing on meaningful features related to robot functionality, situational awareness, data visualization, preferred communication formats, and decision-support needs. Both frequently mentioned items and less common but technically significant insights were coded.

The resulting codes were sorted into 10 preliminary themes by clustering related codes that reflect shared concerns or priorities across participants. At this stage, a concept chart was used to explore how different codes group together under broader concepts. Candidate themes were reviewed for internal consistency and relevance to the research question. Themes were revised or combined based on coherence, with attention to contradictory or divergent views, resulting in six main themes. Each theme was then refined to clearly articulate its central idea, scope, and contribution to the broader understanding of data communication in mine rescue scenarios. Twenty-eight sub-themes were created where useful, for example, to distinguish between preferred data types (e.g., gas levels, robot location) and preferred formats (e.g., audio alerts, visual cues).

1. Mapping theme, spanning passability and geometry, hazards and stability, water and terrain, ventilation and airflow, people and assets, and map alignment, was informed by codes such as hazard reconnaissance, environmental monitoring, and map familiarity, reflecting the priority participants placed on clear, up-to-date spatial information.
2. Gas and environmental data theme, encompassing gas measurement and priority, visualization and thresholding, anomalies and effects, and thermal/fire cues, drew on codes like gas type prioritization, temperature as a hazard indicator, and color-coded safety alerts, emphasizing the need for easily interpretable hazard indicators.
3. Trust and reliability theme, which includes evidence and credibility, trust posture, control and overrides, safe behavior, readiness and endurance, resilience and recovery, and access capability, were supported by codes such as status transparency, human-in-the-loop control, and manual override capability.
4. Update frequency and autonomy theme, covering update cadence, mission bounding, and return/stop conditions, was shaped by codes including fixed update intervals, event-triggered autonomy, and destination-bounded operation.
5. Interface appearance theme, organized around data layers, alerts and cues, layout patterns, and visualization and readability, aligned with codes such as simplicity of design, single-screen visibility, and clear labeling.
6. Human input and map editing theme, incorporating editing and annotation, reconciliation and versioning, role and control preference, and command authority, was informed by codes related to map-editing control, manual input capability, and operator empowerment.

Table 2 shows those emerging themes and their corresponding subthemes.

**TABLE 2:** Themes and subthemes derived from participating interviews.

No	Theme	Subtheme
1	Mapping	Hazards and Stability
		Map Alignment
		Passibility and Geometry
		People and Assets
		Ventilation and Airflow
		Water and Terrain
2	Gas and Environmental Data	Anomalies and Effects
		Gas Measurement and Priority
		Thermal/Fire Cues
		Visualization and Thresholding
3	Trust and Reliability	Access Capability
		Control and Overrides
		Evidence and Credibility
		Readiness and Endurance
		Safe Behavior
		Trust Posture
4	Update Frequency and Autonomy	Mission Bounding
		Return/Stop Conditions
		Update Cadence
5	Interface Appearance	Alerts and Cues
		Data Layers
		Layout Patterns
		Visualization and Readability
6	Human Input and Map Editing	Command Authority
		Editing and Annotation
		Role and Control Preference

### 4.2 Cognitive model integration

The six themes derived from the interviews reveal not only what information mine rescue personnel need from robotic systems, but also how they prefer to receive and interact with that information. To translate these empirical findings into actionable, theoretically grounded design guidance, we interpret them through four established cognitive models: (SA) (Endsley, 1995), (MRT) (Wickens, 2008), (CLT) (Sweller, 1988), and (MM) (Norman, 2002). This analytical step bridges the gap between descriptive results and prescriptive design recommendations. In the following section, we map representative participant quotes to these cognitive frameworks, and in the subsequent discussion, each theme is revisited with its cognitive interpretation to derive targeted, human-centered interface design strategies.

Each excerpt was examined for alignment with these definitions and assigned to the most relevant model(s), with multi-tagging permitted when a statement clearly reflected more than one cognitive mechanism. This mapping did not influence the coding or theme generation stages; rather, it provided a structured, theory-driven interpretation of empirical findings. By situating participant priorities—such as threshold-based gas alerts, layered mapping with editable hazards, and event-triggered notifications—within established cognitive frameworks, we were able to explain why these preferences reduce cognitive load, enhance situational awareness, and improve trust and control in high-stakes rescue environments. This approach strengthened the link between field-derived insights and human factors theory, enabling the derivation of targeted, generalizable design recommendations. Table 3 below presents how the selected cognitive models align with quotes expressed by mine rescue personnel during interviews.

**TABLE 3:** Cognitive models used post-analysis to interpret participant quotes.

Cognitive model	Operational definition (applied in analysis)	Representative quote	Participant role
Situational Awareness (SA, Level 1 and 2) (Endsley, 1995)	Perceiving, comprehending, and projecting environmental hazards and operational conditions to support decision-making. Recognizing and responding to environmental hazards	“I need to know water, gases are a huge thing, low O_2_, high CO, and heat sources … ”	Company superintendent
Cognitive Load Theory (CLT) (Sweller, 1988)	Reducing extraneous mental effort by prioritizing and structuring critical information in a clear, manageable format. Prioritizing critical information to prevent overload	“Everything on one screen would be too much … section by section is better.”	Mining engineer
Mental Models (MM) (Norman, 2002)	Aligning system behavior and interface elements with user expectations and prior experience to foster trust and reduce errors. Building trust through consistency with prior experience	“… once it [robot] proves itself… I want to see the trials and make sure it was actually accurate.”	Director of mining and industrial extension
Multiple Resource Theory (MRT) (Wickens, 2008)	Distributing information across visual, auditory, and other sensory channels to minimize interference and improve multitasking	“Use audio beat, color-coded screen, and thermal scan … ”	General Engineer

## 5 Design recommendations

The thematic findings have shown that participants’ perspectives are deeply shaped by operational realities, mental models built from field experience, and cognitive constraints under stress. Using SA, MM, CLT, and MRT as analytical frameworks (technical), the necessity of delivering information in specific formats, rhythms, and structures to support effective decision-making was discussed. We then achieved a carefully crafted synthesis of design considerations and recommendations that should shape the development process and production of human-robot systems for underground mine rescue operations.

From these themes, we derived six distinct design recommendations, organized into 40 individual design recommendations corresponding to the six themes. It is worth mentioning that, in multiple parts of this discussion, some quotes were intentionally repeated. These statements encapsulate key participant concerns that intersect multiple cognitive demands. By examining them through utilized theoretical lenses (i.e., MM, CLT, MRT, and SA), their layered significance becomes clearer. This approach reflects the richness of participants’ perspectives and strengthens the design recommendations for a cognitively sustainable interface.

### 5.1 Mapping recommendation - Familiarity and functionality

Participants emphasized the need for detailed, editable, and familiar maps that accurately mirror the underground environment in real time. One participant stressed, “I need to know water, gases are a huge thing, low O_2_, high CO, and heat sources” (subthemes of ventilation and airflow). Another reinforced, “It should show roof falls, ventilation doors, stoppings, obstacles” (subthemes of hazards and stability; passability and geometry). These concerns appear interlinked: the environmental hazards (gases, water, heat) and physical infrastructure (roof conditions, doors, barricades) together create a complex risk landscape that responders should simultaneously monitor. This holistic situational awareness is critical, as responders rely on MM that seem to be built through personal field experiences to anticipate hazards and navigate safely. The demand for infrastructure information such as “… door [is] open or closed … ” “roof condition,” “broken, sagging, collapsed areas,” and “obstructions on both sides” (subtheme of subthemes of hazards and stability), confirms the mapping system should be cognitively dense yet visually manageable. This emphasis on needing detailed yet manageable representations of diverse elements, ranging from environmental hazards to structural conditions, strongly imply these recommendations. In interface and spatial visualization design, when multiple types of information should be presented simultaneously without overwhelming the user, it is common to organize such data into overlayers. These layers, often toggleable or stacked, allow users to selectively view relevant categories (e.g., gas levels, heat sources, structural integrity) and reduce visual clutter. Thus, although the term “layer” was not used by participants, this interpretation is grounded in their stated needs and aligns with established visualization strategies for maintaining clarity and cognitive efficiency under pressure. This interpretation aligns with the SA framework, which emphasizes that “situation awareness is enhanced when displays provide integrated information that supports comprehension and projection, rather than requiring the operator to integrate large amounts of raw data mentally. “In addition, participants emphasized including familiar elements on maps, such as “safe entry [points]”, “barricades, refuge chambers, and show water lines” (subthemes of people and assets; water and terrain) because these reflect their established mental models formed during their previous mine safety training and operational experience. Thus, when maps mirror the formats, zones, and indicators that participants are trained to recognize, then, they can quickly interpret and act on the information, reducing the need for additional cognitive effort to translate unfamiliar symbols or layouts. This alignment improves the accuracy and speed of decision-making in urgent situations. These recommendations are consistent with human-factors research on display integration and spatial information design. Wickens and Carswell’s Proximity Compatibility Principle (Wickens and Carswell, 1995) suggests that integrating data layers is only beneficial when the user should mentally combine them; otherwise, separating or toggling layers can reduce interference and improve task performance. Endsley and Jones (Endsley, 2011) further emphasize that layered displays can enhance situational awareness rather than overwhelming the operator. Recent cartographic and HCI work also shows that progressive disclosure and uncertainty-aware overlays reduce cognitive load and improve interpretability in mobile or high-stakes map use (Griffin et al., 2024) (Warden et al., 2023).

### 5.2 Gas and environmental data - Action-oriented, not just informational

Gas data, specifically CH_4_, CO, and O_2_ (subtheme of gas measurement and priority), were identified by nearly all participants as the highest priority. One put it bluntly: “if you don’t have air quality numbers, the rest of it is irrelevant.” This underscores that air quality is the immediate, non-negotiable safety concern underground, and without it, other information holds little operational value. Another explained the connection between gas and projection: “if I knew I had a fire, I knew I had a high CO … fire gases don’t mix … then I can come sit down with a map in my team and say, here’s what you got.” (subtheme of thermal/fire cues). This reveals how participants use gas readings not only as current data but as a basis for mental simulation and forecasting, enabling informed strategic planning. These previously mentioned statements again demonstrate SA, where gas patterns are not passively consumed but mentally simulated into likely scenarios.

To support this, participants requested that systems show “trigger levels,” “explosive limits on monitor,” and “color codes … red for danger, green for safe.” One participant elaborated, “If it hits certain levels, just send the data back—don’t push useless data.” Thus, clear and threshold-based indicators are essential to enable rapid comprehension and action in high-stress environments, where detailed data parsing is impractical. This aligns with CLT: in high-pressure contexts, unfiltered streams of data increase workload and risk. Interfaces should prioritize gas types based on context, filter background information, and generate alerts only when safety thresholds are crossed. Participants also wanted combinatorial awareness: “gas is one, next would be water issues,” and “the thing we do is turn off all the power on the ground. So, water is built everywhere, travel ways, ventilation [airways]…” This shows the importance of designing multi-variable interpretation, where interfaces should support intersections of gas, water, and ventilation conditions. Auditory alerts also came up frequently: “it could have just a beep to get our attention, but it doesn’t have to be a continuous alarm,” and “use audio beat, color-coded screen, and thermal scan.” These preferences are consistent with MRT, as different sensory channels are used for different urgency levels. Taken together, these preferences point to a need for robot systems interfaces that are threshold-driven, context-aware, and multisensory to enable quicker decisions under pressure.

The proposed use of threshold-based, color-coded hazard indicators aligns with research showing that urgency-based color schemes improve the speed and accuracy of hazard detection under time pressure. Studies in aviation and process control have found that well-designed color coding allows operators to make rapid go/no-go decisions without parsing raw data. Safety standards such as ANSI Z535 also specify consistent use of red, amber, and green to communicate danger, caution, and safe states, supporting the recommended palette for gas monitoring interfaces (Murphy et al., 2009; Friedrich and Vollrath, 2022; Blundell et al., 2020).

### 5.3 Trust and reliability-transparency, experience, and control

Trust in robotic systems was conditional and shaped by accumulated field experiences. “… once it proves itself, I want to see the trials and make sure it was actually accurate” (subtheme of evidence and credibility), one participant said. Another was more skeptical: “… no, I don’t trust anything electronic. If it [system failure] only happens one percent of the time, that’s not acceptable.” These statements reflect mental model violations, where the system’s behavior did not align with expectations in past missions. These concerns about trust are grounded in miners’ direct or indirect experiences with real-world failures of rescue robots during past disasters. For instance, during the 2006 Sago Mine disaster, the Wolverine V2 robot from CRASAR became inoperable after its tether became entangled and power was lost within the first 40 feet and also, regarding the Wolverine robot, a single operator was unable to track the robot’s location and operate it simultaneously (Murphy et al., 2009). In the 2007 Crandall Canyon collapse (Teaster and Pavlovich, 2008), a robot provided by the DoD’s Robotics Systems Joint Project Office failed due to dust-related sensor interference and unstable terrain (Teaster and Pavlovich, 2008). In Australia’s Beaconsfield Mine collapse (2006), a robot from the Australian Centre for Field Robotics could not be used at all due to extreme spatial constraints and tunnel irregularities (Reddy et al., 2015). Also operating the Numbat was demanding due to poor lighting conditions and unfamiliar terrain, significantly increasing the operator’s workload and led to mission failure in the testing step. These failures violated users' expectations of robot systems’ reliability and resilience in high-stakes environments and reinforced the mental model violations highlighted by participants. As such, the demand for extensive validation before deployment, as one participant put “once it has a history”, reflects not only theoretical concerns but practical, field-based distrust rooted in prior breakdowns. Participants demanded validation and calibration: “you don’t need to verify it - once it has a history, … once it has a successful mission, then the idea is to use it.” This points to a trust trajectory that can be earned through consistent performance and explainable behavior. CLT supports this: systems that obscure their decision-making increase mental workload and reduce trust. Others emphasized human control: “I want to control the robot’s actions unless I’m desperate,” and “data—yes, decision-making—no” (subtheme of control and overrides). Trust was not about full autonomy, but about shared control, where humans always have final authority. An Interface should display robot confidence levels, error logs, and manual override functions to reinforce confidence without removing oversight.

These findings reflect broader HRI literature showing that trust is improved when systems are transparent, align with user MM, and offer clear avenues for human override. Shared MM has been shown to support faster coordination and error recovery, while transparency and explanations help calibrate trust and avoid overreliance. Reviews in HRI highlight that providing evidence of past performance and allowing operator control are key to sustaining trust in autonomous systems (Tabrez et al., 2020; Ezenyilimba et al., 2023; Almasi et al., 2023).

### 5.4 Update frequency and autonomy-pacing that matches cognitive workflow

When asked about update timing, participants offered rhythms aligned with decision-making cycles: “Every ten to 15 min gives me enough time to think, strategize what I want to do, and then write it up and send it around the table.” Others preferred “every 5 min” for high-risk stages or “every 1000 ft of progress” (subtheme of update cadence). This illustrates that information pacing supports cognitive functions at multiple levels, including situational awareness and the internal coordination of thought within individual rescuers. Thus, this shows the need for user-defined update intervals that match cognitive and operational rhythms which are supported by MRT and SA. Some participants expressed a desire for real-time anomaly detection: “if the robot finds fire, return. If it finds an explosive, return. If it finds a person, return immediately.” This demand for event-based interruption indicates the need for systems that push data based on conditions, not just intervals. Participants also wanted distance-based update options, noting, “tell us what’s there every 10 ft.” Therefore, interfaces should allow users to configure update pacing based on distance, time, or condition triggers, and clearly indicate “time since last update” and “next expected update.” Taken together, these insights point to a need for interfaces that support configurable update logic that balances scheduled intervals with event-driven alerts to match the way rescue teams think, operate and respond underground. The recommendation to combine user-defined update intervals with event-triggered alerts is supported by alarm-management standards (ANSI/ISA-18.2, IEC 62682) and cross-industry human-factors research (International Society of Automation, 2016; Pruitt et al., 2023). These guidelines emphasize prioritizing critical alerts, suppressing non-actionable ones, and tailoring update pacing to operational tempo to reduce alarm fatigue. Applying these principles in mine rescue robotics can ensure that operators receive timely, relevant data without cognitive overload. d5.5 Interface Appearance and Usability - Modular, Hierarchical, and Customizable.

Interface layout was another area of strong consensus. Participants rejected cluttered, flat displays: “all the data in one screen? No—every screen just one data,” and “just show me section by section, every 20 breaks or 1000 ft” (subtheme of layout patterns). Others requested “pop-ups,” “scrollable panels,” or “a home screen that gives it all to you if needed.” Several participants expressed the importance of contextual grouping: “Combine all the data—water, gas, temperature—in one section and flag it red if it’s dangerous” (subthemes of alert cues; data layers). Others requested flexibility: “Some people may like everything at once. I’d prefer to scroll through.” These responses underscore the need for role-based or customizable dashboards, which allow responders to choose between granular or summary views based on their task and stress level.

Research on multimodal alerts demonstrates that combining auditory and visual cues enhances detection rates and decision speed in high-stakes environments. Human-factors studies in driving, aviation, and clinical monitoring show that well-coordinated multisensory warnings reduce missed alerts and speed operator response. Additionally, dashboard usability reviews highlight the importance of customizable, role-based displays to support diverse operator preferences and maintain situational awareness (Almasi et al., 2023; Mehrotra et al., 2022; Burdick et al., 2022; Edworthy et al., 2021).

### 5.5 Human input and map editing-empowerment and shared cognition

Participants are expected to be able to actively shape the information space. “Let me add it … I think there should be something here … compare [the] new map and [the] real map,” one said. Another emphasized, “if they spotted anything like roof fall, we want to add” (subtheme of editing and annotation). These statements reflect SA and MM adaptation, where users interpret new inputs and update their internal and shared representations accordingly. Another participant reinforced, “yes, we want to update [the] mine map based on [the] development map” (subtheme of reconciliation and versioning). These quotes show the importance of interfaces that allow manual annotations, map corrections, and visual synchronization with new hazards, ensuring that evolving situational knowledge is embedded into shared cognition. Systems that auto-update without user review risk violating mental alignment and may erode trust. Interfaces should allow intuitive map annotation, flagging of hazards, and undo/history features to support reflection and collaboration. Overcomplicated editing systems would reduce use during high-stress operations and risk outdated information persisting. The goal should be to create a shared cognitive environment between user and robot, a digital mirror of the underground space that evolves through joint interaction (subtheme of role and control preference). This analysis reveals that mine rescue personnel are not merely data recipients but strategic interpreters, seeking interfaces that fit their cognitive workflows, prior experiences, and operational goals. From real-time maps to gas visualization, from update pacing to manual override, every interface function should align with cognitive theory and field reality (subtheme of command authority).

Allowing operators to annotate and edit maps is consistent with cognitive systems engineering principles, which emphasize externalizing state information to support shared situational awareness. Editable overlays and change-history features enable teams to reconcile evolving field observations with system data, improving trust and accuracy. Recent HCI studies in cartography also show that user-driven, layer-by-layer construction supports interpretability without increasing workload (Endsley, 2011; Griffin et al., 2024).

A summary of interface design recommendations was also presented in Table 4 below.

**TABLE 4:** Participant expectations for mine rescue robots based on interview questions and analytical themes.

Representative quotes	Cognitive models	Recommended design features	Design pitfalls to avoid
Mapping recommendation theme			
• “It should show roof falls, ventilation doors, stoppings, obstacles…” • “Map should include safe entry, barricades, refuge chambers.” “Roof conditions, gas data. All of them at the same time” • “Width, height, where all openings are… if doors are open or closed.” • “Map should include safe entry, barricades, refuge chambers • “Obviously clear passageways. I need to know water. Yeah, gases. Gases are a huge thing. Low O2, high CO… intact stuff, or stoppings intact, waterline intact … ”	SA (1and2) MM	• Use color-coded gas indicators (e.g., red for high CO) to understand severity of gas levels (show unsafe/Safe zones) • Include real-time display of gas data, water presence and environmental hazards helps operators detect life threatening conditions • Provide audio or visual alerts matching training expectations • Create layered dashboard view with toggle-able elements • Use map overlays for stoppings, roof falls • Use icons and layout that mirror physical maps/miner safety docs • Display refuge chamber locations clearly Show door status (open/closed) with icons • Use visual indicators (green = intact, red = failed) for stoppings, waterlines • Display status next to structure names to reduce interpretation load	• Flat maps lacking hazard overlays Missing safety-critical structures (e.g., refuge chambers) • Unfamiliar or non-standard icons/layouts Mixing unrelated visual elements that reduce clarity • Presenting all information at once with no filtering or progressive detail
Gas and environmental data theme			
• “If I knew I had a fire, I knew I had a high CO… So, fire gases don't mix… Then I can come sit down with a map in my team and say, here’s what you got.” • “Low O2, high CO… Those are all the critical things…” • “CH4, CO and O2 are important for me.” • “Influence of drone propeller on gas concentration must be considered.” • “If you don't have air quality numbers, the rest of it is irrelevant” • “Gases are a huge thing. Low O2, high CO… and heat source” • “Methane, CO, O2… probably the methane number” \	SA (1 and 2 and3) CLT MM	• Display linked data trends (e.g., CO + temp = fire) • Prioritize CH4, CO, O2 in the UI • Show gas data with color-coded • thresholds and ranking of danger Include inferred condition alerts (e.g., fire risk) • Allow filtering of non-critical gases • Provide time-stamped history of spikes or danger changes	• visual overload — focus on simplicity, hierarchy, and familiarity • Showing gas data in isolation without thresholds or warning cues • Presenting raw numbers without context or alert logic • Overloading interface with too many gas types equally emphasized Design for interpretation and decision-making, not just data display
Trust and reliability theme			
• itself… I want to see the trials and make sure it was actually accurate.” • “The tethered robot didn’t work well; we had to go around it.” • “We need to test it in dusty/smoky environments.” • “No, I don't trust the robot… we’ve never been successful with a robot yet” • “Drones and robots have to be used in a limited capacity.” • “I have never trust anything electronic”	MM SA, and CLT	• Provide historical logs of robot behavior and performance accuracy enable transparency in robot decisions • Allow user to override or verify decisions Indicate reliability or confidence levels next to system outputs • Show trial/test results with visual badges or trust indicators • Use transparency (don’t hide robot uncertainty) • Design for shared control • Match interface behavior to what users expect from past experience (accurate, explainable, limited autonomy) • Build trust gradually: provide evidence, transparency, and accurate feedback • Structure names to reduce interpretation load	• Full autonomy without feedback or human control • Black-box system behavior • Suppressing alerts or hiding failed performance • Overburdening users with unexplained outputs • Don’t remove human-in-the-loop mechanisms
Updated frequency and autonomy theme			
• “Robots move through slow… I’ve got to put boots on the ground… Time is of the essence… clock’s ticking… 96 h” • “I want it to update every five to 10 minutes… longest 15–20 min” • “Push back data as fast as you can.” • “Prefer setting a destination rather than a time limit.” • “if you find a person return immediately if you find explosive return if you find fire, get back in communication else if back in … Min ((I am going to pick my mission option from commanders’ critical information)” • “We want to go 1000 ft every time and check every ft of that” • “Every 10–15 min (So every 10 minutes gives me enough time to think, strategize what I want to do. And then 10 minutes to write it up and send it around the table)” “15 min (15 30 45 1-h increments) (depend where we are on the time scale or the timeline of the uh of the event”	MRT SA, and MM	• Support adjustable update intervals (e.g., 5–15 min Show time since last update • Use countdown timer to next update • Allow real-time push for critical data • Provide multimodal alerts (text + sound + visual) • Let users switch between manual and auto-update modes • Show mission clock prominently • Provide distance trackers, waypoints, and scanning logs per segment • Match update frequency to user-defined cognitive rhythm (10–15 min), allow pause-and-review functions Reflect field logic (e.g., distance-based goals, event-driven decisions, familiar procedures)	• Static update frequency in dynamic operations (Don’t present time-insensitive data in time-critical situations) • Inconsistent update intervals • Delaying critical info behind scheduled refreshes • Don’t skip areas; don’t allow jumps in data without user confirmation Don’t overwhelm with constant data avoid “one-size-fits-all” intervals avoid forcing rapid decision-making
Interface appearance theme			
• “It would be nice if it sent it back programmable to a computer… look at a Visio map… SPAD numbers… correlate… point of reference…” • “Explosive limits on monitors and screens that light up (some sort of light, red light, strobe light, probably light of some kind to catch, oxygen between X and Y, if it’s in there, it’s green. Below or above in red. Same for methane, Red is always. Good. So, when it travels, if it can send a green signal. It’s all good. Should let us know about low o2, high co” • “Audio beat, it could have a just a beep to get your attention but it doesn't have to be a continuous alarm continuous alarms tend to be very distracting.” • “Like to have pop ups” • “One screen you can have a home screen that gives it all to you. Some people may like that. I would prefer to be able to see, here’s what my gases are. Here’s how it maps the terrain. Here’s what the temperature is. That’s how I would like to see it. Just like scrolling off the front. Here at… Every screen,” just one data,” • “But I want X amount of breaks at a time. It’ll do 20 on a screen. I’m going up a panel.? That section should be like thousand feet. 20 breaks. At 80 feet on center breaks. So, 1600 feet I want you to do it in section because I will travel with my team by section, you want to show data like section by section. The information here. The gas concentration. Everything related to that section”	MRT SA, and MM	• Enable customizable home/dashboard view • Use color, light, and sound to convey gas thresholds, explosive risks, and robot status • Provide toggle between summary and detailed views • Keep each section (gas, map, status) modular • Allow scrollable or swipe-based info access • Provide visual hierarchy • Present section-based views (e.g., every 20 breaks or 1000 ft)	• Overloading a single screen with too much info • Using abstract layouts unfamiliar to field users • No separation between unrelated data types • Avoid constant beeping alarms (don’t make all alerts equal in intensity)
Map and editing human input			
• “Let me add it… I think there should be something here … edit the map… compare new map and real map… So if you have roof wall everything you can just compare … ” • “Yes, we want to update [the] mine map based on [the ]developed map”	MM, SA, and CLT	• Enable manual annotation of maps • Allow flagging of roof falls, collapses, or added wall • Use visual change detection tools (e.g., overlays) • Include undo/history features in map editing	• Locking maps from user input • Auto-updating maps without user review • Overcomplicated editing workflows that slow down use in time-critical missions update

Equally important are the design mistakes to avoid. Thus, interfaces should not:

1. Present raw, unfiltered data in cluttered layouts
2. Force users to mentally combine information from scattered displays
3. Automate decisions without offering manual override
4. Hide system confidence or failure indicators
5. Use unfamiliar symbols, non-editable maps, or rigid update intervals

**FIGURE 3:**
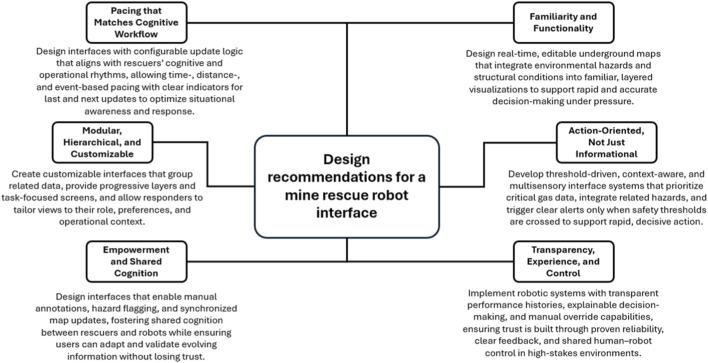
Key Recommendations for designing an intuitive interface for mine rescue robots.

These violations lead to mistrust, increased error risk, and loss of situational control—especially in emergencies where every second matters. Designing for search and rescue robotic systems is not just about sensors and autonomy—it is about supporting the human mind in one of the world’s most dangerous environments. Thus, this study offers a foundation for building such systems by placing expert users at the center of every interface decision, as shown in Figure 3.

## 6 Discussion

Despite the valuable insights generated by this study, some limitations should be acknowledged. First, the sample size was relatively small. While this group provided deep and field-grounded expertise, the findings may not fully capture the diversity of perspectives found in other mining regions, operational contexts, or organizational cultures.

Second, the results are context-specific, reflecting the unique cognitive and environmental demands of mine rescue. While some design recommendations may be transferable to other domains involving human-robot interaction in high-risk environments, caution should be exercised in generalizing these findings beyond the immediate context of underground mining emergencies. Furthermore, this study relied on qualitative interviews, focusing on user perceptions, preferences, and retrospective accounts rather than direct observations or empirical testing of interface performance. As such, the cognitive alignment and design recommendations identified here require further validation through applied experiments, simulations, or field trials.

In particular, further research is needed to deepen and broaden the six thematic categories identified here—mapping, gas and environmental data, trust and reliability, update frequency and autonomy, interface appearance, and human input and map editing—across different mine types, organizational structures, and technology levels. The design recommendations proposed in this study should be empirically verified by embedding them in prototype interfaces and evaluating their impact on situational awareness, workload, decision-making quality, and team coordination in realistic simulations, training exercises, and field trials. Such multi-method validation will clarify which recommendations generalize, which require adaptation, and where additional cognitive or domain-specific factors should be incorporated.

Finally, while features like real-time mapping and adaptive alerts are highly desirable, their implementation is limited by technical constraints. Underground environments often lack reliable high-bandwidth communication, making real-time data transfer difficult this is precisely why configurable update intervals become essential, allowing systems to balance data delivery with connectivity constraints.

## 7 Conclusion

This study offers an in-depth exploration of human-robot information exchange in underground mine rescue operations. Grounded in structured thematic analysis and informed by cognitive models, the findings move beyond general recommendations to offer targeted and field-validated design strategies. Through interviews with experienced mine rescue personnel, several core themes emerged: the need for real-time maps, gas and hazard alerts that support action—not just awareness, flexible update pacing, intuitive visual layouts, and conditional trust in robotic systems and users’ input. These insights reflect not only practical concerns in the field but also deeper cognitive demands for filtering, focus, and control under extreme pressure.

Participants consistently stressed that interfaces should function as cognitive partners, not passive data screens. Information should be filtered and contextually prioritized to support decision-making. Systems should highlight danger thresholds, synchronize with operational rhythm, and visualize spatial and environmental risks in formats responders already understand. This implies a design strategy rooted in modular displays, editable map layers, and multimodal alerts (e.g., sound + color), all tailored to reduce mental workload and improve comprehension.

Trust and autonomy should be earned, not assumed. Users expressed a strong desire to retain control, verify information, and contribute field knowledge through manual edits and annotations. Interfaces that prevent user input or fail to reflect evolving field conditions risk becoming irrelevant or even hazardous. In conclusion, this study provides a practical framework for designing cognitively aligned human-robot interfaces. By incorporating shared cognition, flexible control, familiar visual elements, and alert prioritization—while avoiding complexity, rigidity, and automation-overreach—developers can create systems that potentially enhance mine rescue effectiveness, safety, and human-robot synergy when it matters most.

Future research should focus on implementing the identified design recommendations into a functional prototype interface tailored for underground mine rescue operations. The next step should involve usability testing with mine rescue personnel under simulated and controlled conditions. These tests would help evaluate the effectiveness of features such as layered maps, danger-triggered alerts, flexible update pacing, and manual override controls. Iterative improvements should be driven by expert feedback, with particular attention to optimizing real-time data access, reducing cognitive load, and improving interface clarity. Such evaluations will be essential for developing user-centered, cognitively aligned robotic tools that enhance situational awareness, decrease workload, improve decision-making, and operational trust in high-stress rescue environments.

## Data Availability

The raw data supporting the conclusions of this article will be made available by the authors, without undue reservation.

## Appendix A

### Semi-structured interview guide

#### Opening questions

- May I ask what your current job title is?
- How long have you been working in the mining industry?
- Have you ever been in a search and rescue operation?

### Section 1: mapping

1. If a robot could provide detailed, real-time maps, what specific information would you like to see (e.g., obstacles, tunnel layouts, escape routes)?
2. Do you think having other information next to the map (for example, gas information, robot positioning, etc.) would be helpful or distracting?
3. Do you want to edit the map as you go forward? How?

### Section 2: navigation

1. If providing real-time information is not possible, do you trust the robot to navigate to your desired destination autonomously? What kind of time frame are you expecting to get that information back to make a decision?
2. Do you prefer to use destination limit or time limit for the robot to explore an area on its own? Why?

### Section 3: gas sensor

1. If the robot can collect gas data for the mine rescuers, how do you want to see the robot visualize the data? Which gases data has priority for you? Do you need other information besides gas map to make decisions?
2. If real time transfer of gas data is not possible, what is the acceptable latency for gas data?
3. How would you expect the robot to alert the team when it detects hazardous gases, and what kind of response or action should follow this detection? Would you prefer visual or auditory alerts when certain threshold levels are detected by the robot?
4. What specific data do you want to see first, and how would you prioritize its importance for decision-making?

### Section 4: communication

How can the robot’s 4 (for both control and data visualization) be designed to ensure ease of use under stress, which is common in emergency situations?

### Section 5: robots role and responsibility/reliability and control

1. What role do you think robots should play interacting with mine rescue? Tool, guide or partner?
2. What tasks do you think robots should handle entirely by themselves, and which ones should remain under human control?
3. Would you prefer a robot that learns from previous missions and adapts its behavior, or would you prefer a static set of instructions?
4. How much do you trust the robot’s decision-making in complex rescue operations? Are there specific actions you would always want to oversee directly?
5. How should the robot tell you when something goes wrong? Should it suggest solutions or just give you an alert?
6. What features would make you feel confident relying on the robot for high-risk missions? (Redundancy system, real time updates, proven reliability, emergency override, …)
7. Will you take gas data even if the robot collects those data?
8. Do you think command center should have the ability to override the decision by the mine rescuers based on robot data?
9. Would you want the robot to prioritize data collection over returning to base if its battery life is low, or should it prioritize coming back safely?
10. In case the robot makes an error or malfunctions, how do you think it should recover?
